# Prevalence and Predictors of Iron Deficiency Anemia in Children under Five Years of Age in Pakistan, A Secondary Analysis of National Nutrition Survey Data 2011–2012

**DOI:** 10.1371/journal.pone.0155051

**Published:** 2016-05-12

**Authors:** Muhammad Atif Habib, Kirsten Black, Sajid Bashir Soofi, Imtiaz Hussain, Zaid Bhatti, Zulfiqar A. Bhutta, Camille Raynes-Greenow

**Affiliations:** 1 Discipline of Obstetrics, Gynaecology and Neonatology, Central Clinical School, University of Sydney, Sydney, New South Wales, Australia; 2 Women and Child Health Division, Aga Khan University, Karachi, Pakistan; 3 Sydney Medical School, Sydney School of Public Health, University of Sydney, Sydney, New South Wales, Australia; National Institute of Agronomic Research, FRANCE

## Abstract

**Background:**

Iron deficiency Anemia (IDA) in children is a recognized public health problem that impacts adversely on child morbidity, mortality and impairs cognitive development. In Pakistan information on the true prevalence and predictors of IDA is limited. This study sought to investigate IDA in children under five years of age using data from a nationally representative stratified cross-sectional survey.

**Methods:**

Secondary analysis was performed on the National Nutrition Survey in Pakistan 2011–2012. We used a pre-structured instrument to collect socio demographic and nutritional data on mothers and children. We also collected Anthropometric measurements and blood samples for micronutrient deficiencies. IDA was defined as having both haemoglobin levels of <110 g/L and ferritin levels of < 12 μg/L. Data analysis was performed by applying univariate and multivariate techniques using logistic regression through SPSS.

**Findings:**

A total of 7138 children aged between 6–59 months were included in the analysis. The prevalence of IDA was 33.2%. In multivariate regression analysis adjusted odds ratios (AOR) were calculated. Age < 24 months (AOR 1.40, 95% CI 1.18–1.55 p <0.05), stunting (AOR 1.42 CI 1.23–1.63 p<0.05), presence of clinical anemia (AOR 5.69 CI 4.93–6.56 p<0.05), having a mother with IDA (AOR 1.72 CI 1.47–2.01 p<0.05) and household food insecurity (AOR 1.20 CI 1.10–1.40 P<0.05) were associated with IDA. Living in a rural area (AOR 0.77 CI 0.65–0.90 p<0.05) and being a female child (AOR 0.87 CI 0.76–0.98 p<0.05) were associated with reduced odds of IDA.

**Conclusion:**

The prevalence of IDA amongst Pakistani children represents a moderate burden that disproportionately affects the youngest, growth retarded children, affected children are more likely to have mothers with IDA and live in areas where food security is lacking. National efforts to alleviate the burden of IDA should involve both short term vertical programs such as iron supplementation and long term horizontal programs including wheat flour fortification.

## Introduction

Iron deficiency anemia in young children is recognized as a major public health issue and the most prevalent form of micronutrient deficiency worldwide [1, 2]. The global prevalence of anemia (defined as hemoglobin level of <110 g/L) in children aged 6–59 months is 43% and half is attributable to iron deficiency anemia (IDA) which is defined as hemoglobin level of <110g/L and ferritin level of < 12 μg/L [1–4]. IDA contributes substantially to childhood mortality and morbidity and is linked to impaired brain development and cognitive functions [5–9]. IDA is also ranked as the third leading cause of disability worldwide and the 13^th^ leading risk factor for the global disability adjusted life years [10]. Most of the burden of IDA is in the resource poor settings of Africa and Asia [4, 11].

In Pakistan the reported prevalence of IDA in children under five is between 40–70% [12–16]. In Pakistani children IDA has been associated with growth retardation, impaired cognition, reduced physical activity and postulated as a contributor to the high national infant mortality rate [10, 12, 14, 17, 18, 19]. Widespread micronutrient deficiencies along with other clinical and social factors are believed to be the leading cause of IDA in Pakistan [13–18]. However prevalence data is scarce with many studies more than a decade old, or based on small numbers and in small non-representative populations. Further, IDA is best defined as the combination of both low hemoglobin and low ferritin concentrations, whereas the published studies to date from Pakistan are mostly based solely on hemoglobin concentrations.

As such, nationally representative robust data are still lacking to determine the socio-demographic factors associated with IDA that will enable the development of local strategies to treat and prevent IDA in Pakistani children. In this study we aimed to estimate the prevalence of IDA in Pakistani children and to evaluate factors associated with IDA by conducting a secondary analysis of the Pakistan National Nutrition Survey that was undertaken between 2011–2012.

## Materials and Methods

### Data source

The data used for this analysis were derived from the Pakistan National Nutrition Survey 2011–2012. The survey was conducted by the Aga Khan University (AKU) in collaboration with the Federal Ministry of Health in Pakistan and was funded by UNICEF. The survey was a stratified representative cross-sectional national survey with provincial specificity, with a two-stage stratified sampling design. The sampling frame in the form of Enumeration blocks (1500 in number) was provided by the Pakistan Federal Bureau of Statistics. Each enumeration block was demarcated, mapped and listed before the actual data collection and from each enumeration block 20 households were selected randomly through a computer generated program. A total of 30,000 households were included, 12,360 were urban households and 17,640 were rural, resulting in 27,963 respondents. Households with children between the ages of six months to five years were included in the survey. One married woman of reproductive age (15–49 years) with at least one child aged less than 5 years was chosen from each selected household. If there were multiple eligible women in a household, one woman was randomly selected.

A pre structured and pre tested instrument was used for data collection. The instrument collected data on socio-economic status, maternal reproductive history, child medical history, food security, anthropometric measurements and biochemical measurements of various micronutrients. For anthropometric measurements weight was measured using lightweight SECA scales designed and manufactured under the authority of the United Nations Children’s Fund (UNICEF) and the height/lengths was measured by height boards made by Shorr Inc. Children under 2 years of age were measured lying down on the board and standing height was measured for older children [20].

In children more than 2 years height-for-age z scores (HAZ), weight-for-age z scores (WAZ), weight-for-height z scores (WHZ) were calculated and for children less than 2 years length was used instead of height to calculate length-for-age z scores (LAZ) and weight-for-length z scores (WLZ). Body Mass Index (BMI) was calculated for mothers using height and weight. Children were reported as stunted, wasted or undernourished and mothers were reported as underweight, overweight or obese as per the WHO classification [21]. Blood samples for hemoglobin measurement and micronutrient deficiencies were collected from one child in each household. Venous blood was collected and serum was separated within half hour of collection, using portable centrifuge machines with backup power. The hemoglobin was measured from the venous blood at field while the serum samples were transported to nutrition research laboratory at Aga Khan University under cold chain conditions through country wide network of AKU laboratories collection centres.

Serum Ferritin concentrations were measured for iron deficiency because it has the highest sensitivity and specificity to detect iron deficiency in individuals [22]. The ferritin levels were adjusted for alpha (1)-acid glycoprotein (AGP) and C-reactive protein (CRP) to take into account that ferritin is an acute phase protein and is raised in inflammatory conditions [23–24]. Serum Ferritin was measured by electro-chemiluminescence immunoassay using Roche Cobas E41 Chemistry Analyzer and hemoglobin concentration was measured in whole blood using a Micro cuvette containing a dry reagent system and a dual wavelength HemoCue 201 Photometer. Hemoglobin concentration was also adjusted for altitudes of more than 1000 meters using the WHO recommended altitude formula [25]. Blood samples were also analyzed for zinc deficiency and vitamin A deficiency. Serum was used to estimate the zinc and vitamin A deficiency and the samples were analyzed using Spectrophotometric method for zinc deficiency and Reversed Phase Chromatography method for vitamin A deficiency.

All survey activities were monitored to ensure the data quality. The questionnaire was pre-tested prior to implementation in the field and a pilot survey was also conducted. Competency of field staff was also taken in account prior to hiring and pre-test and post-test was conducted for all field staff during trainings. The survey employed internal and external data monitors to ensure data quality with respect to the all the procedures and survey activities. The anthropometry and equipment HemoCue was calibrated daily for any possible errors. The team leaders analyzed the data (plausibility checks and digit preference) using ENA Smart software and provided regular feedback for improvement. Similar quality assurance steps were duly considered during data entry and cleaning.

Ethical approval for the survey was obtained from the Ethical Review Committee of Aga Khan University and the National Bioethics committee of Pakistan. Written informed consent was obtained from the mothers of the selected child. In case of illiterate mother, consent was documented by a thumbprint on the consent form and a signature by a literate witness. All the names and personal information regarding the participants were kept confidential and data set was kept anonymous for analysis.

### Description of Variables

Child IDA status was the main outcome variable and was defined as the having the combination of haemoglobin levels of <110 g/L and ferritin levels of < 12 μg/L [26]. The selection of explanatory variables for analysis was informed by the literature and their availability in the dataset and is fully described in Table 1. Variables were grouped into three categories; household, maternal, and child. Under the household category area of residence which was categorized into rural and urban, socioeconomic status which was ascertained by computing the wealth quintiles (from being poorest to wealthiest) using the standard demographic and health survey tool and food security status using the standard household food insecurity access scale developed by Food and Nutrition Technical Assistance (FANTA) project [27] and categorized as (Food Secure and Food insecure) were considered. In the maternal category maternal education which was defined as years of education completed (illiterate/years of education), maternal IDA estimated on the basis of low hemoglobin (< 120 g/L) and low ferritin (< 12 μg/L) concentrations and maternal BMI calculated on the basis of height and weight of mother and categorized as <18.5 underweight, 18.5–24.99 normal weight, 25–34.99 overweight/obesity and ≥35 severe obesity, were examined. In the child variables sex, age, clinical anemia estimated through physical examination of conjunctiva, vitamin A deficiency defined as serum vitamin A concentration of < = 0.70 μmol/L and categorized as deficient and non-deficient, zinc deficiency defined as serum zinc concentration of <60 μg/dL and categorized as deficient and non-deficient, stunting defined as height/length-for-age z-score of < −2, wasting defined as weight for height z-score of < −2, underweight defined as weight for age z-score of < −2 as per and history of worm infestation ascertained through recall of worm infestation in last six months.

**Table 1:** Description of Explanatory Variables used in the Analysis.

Variables	Description
**Household level factors**	
Area of residence	urban and rural
Socio economic status of the household	SES was measured as quintiles of a linear index derived from household assets and utilities score, the wealth quintiles were divided into five (poorest, poorer, middle, richer, richest)
Food security status	Food security status was measured by using the standard household food and hunger scale developed by Food and Nutrition Technical Assistance (FANTA) project and categorized as (Food Secure and Food insecure)
**Maternal Factors**	
Maternal Education	Years of education completed (illiterate/years of education)
Maternal Iron deficiency anemia status	Hemoglobin levels of <120 g/L and serum ferritin levels of <12 μg/L. Categorized as Iron deficiency anemic and normal
Maternal BMI	Calculated on the basis of height and weight of mother and categorized as <18.5 underweight, 18.5–24.99 normal weight, 25–34.99 overweight/obesity and ≥35 severe obesity.
**Children factors**	
Sex	Male, female
Age	Categorized as <24 months, 24–59 months
Anemia	Hemoglobin concentration of <110 g/L in whole blood and characterized as anemia
Clinical Anemia	Presence of anemia on physical examination i-e conjunctival and palmar pallor and categorized as Yes or No
Vitamin A Deficiency	Serum vitamin A concentration of < = 0.70 μmol/L and categorized as deficient and non-deficient
Zinc Deficiency	Serum zinc concentration of <60 μg/dL and categorized as deficiency
Height for Age–Stunted	Height/length-for-age z-score of < −2 as per WHO standards and categorized as stunted
Weight for Height—Wasted	Weight for height z-score of < −2 as per WHO standards and categorized as wasted
Weight for Age—Under weight	Weight for age z-score of < −2 as per WHO standards and categorized as underweight
History of worm infestation	History of worm infestation in the previous six months and categorized as Yes or No

### Statistical Analysis:

All data analyses were conducted in IBM SPSS version 19 [28] using a complex sample procedure to allow for adjustments of the sampling design implemented in the survey. The frequencies, along with weighted percentages, were reported for selected predictors and the mean with 95% confidence intervals (CIs) were calculated for the outcomes. The analysis started with simple univariate analysis followed by multivariate logistic regression. Unadjusted odds ratio with their 95% CIs were reported for the bivariate analysis. Variables significant at p<0.25 were considered for inclusion in the multivariate model. Covariates that were insignificant at the multivariate level were dropped consecutively from the model after careful assessment of confounding. The final model was selected on the basis of theoretical and statistical significance of predictors. The Type 1 error rate was set at 0.05. The model estimates are presented with the adjusted odds ratios and 95% CI.

## Results

A total of 7138 children aged between 6–59 months were included in the analysis among them 2373 (33.2%) children had IDA based on low hemoglobin and ferritin levels. Analyses of the child blood samples found that overall (4264) 62.3% were anemic (hemoglobin concentrations <110 g/L), of those who were anemic (283) 4.1% were severely anemic (< 70 g/L) and (3981) 58.3% were moderately anemic (70 g/L – 109 g/L). Ferritin deficiency was identified in (3361) 47.1% children. Among those who had IDA the mean values for hemoglobin and ferritin concentrations were 70 g/L and 6μg/L respectively. The prevalence for vitamin A deficiency and zinc deficiency was 52.6% and 38.8% respectively. Clinical anemia was found in 30.1% children. The anthropometric data identified 44.5% of the children as being stunted, 15.5% as wasted and 33.6% as underweight.

Among maternal factors the prevalence of IDA was found to be 20.0%, which was determined by using maternal hemoglobin and ferritin levels, and according to the maternal anthropometrics 16.0% of mothers were classified as underweight, 21.2% overweight and 11.1% obese. Among all children included in the analysis the majority of children came from rural areas (70.1%), 48.3% of children were female and 47.9% were under the age of 24 months. The two lowest wealth quintiles accounted for 43.3% of the study population, and the two highest was 36.0%. Of the sampled households 64.3%were food insecure. Only 8.3% of the mothers reported that their child had worm infestation in last six months (Table 2).

**Table 2:** Background characteristics of sampled children (6–59 months) in National Nutrition Survey Pakistan (N = 7138).

Background characteristics	N	(%)	95% CI
Residence			
Rural	4331	70.3	66.6–73.2
Urban	2807	29.7	26.8–33.4
Wealth quintiles			
Poorest	1533	23.0	20.4–25.3
2	1390	20.3	18.35–22.0
3	1500	20.8	19.1–22.6
4	1452	19.4	18.1–21.7
5 wealthiest	1263	16.6	14.3–18.4
Food security			
Food insecure	4339	64.3	62.2–66.6
Food secure	2615	35.7	33.4–37.8
Maternal Education			
Illiterate	4015	58.0	54.8–60.1
Primary or less (1–5 years of completed schooling)	1018	14.7	13.6–16.7
Middle(6–8)	605	8.3	7.3–9.7
Matric(9–10)	815	10.8	9.6–12.3
Intermediate & above (>10)	640	8.3	6.7–9.5
Maternal iron deficiency anemia Status			
Anemic	1212	20.0	19.8–23.4
Normal	5127	80.0	76.6–80.3
Maternal BMI (kg/m2)			
<18.5 (Underweight)	1117	16.0	14.9–17.8
18.5–24.9 (Normal)	3660	51.7	49.7–63.8
25–29.9 (Overweight)	1543	21.2	19.3–22.7
> = 30 (Obese)	818	11.1	9.6–12.1
Sex			
Female	3439	48.3	45.8–49.8
Male	3699	51.7	50.3–54.2
Age in months			
24-59m	3755	52.1	47.2–51.3
<24m	3383	47.9	48.7–52.8
Presence of clinical Anemia			
Yes	2057	30.1	33.1–37.7
No	5022	69.9	62.4–66.9
Hemoglobin Levels			
Anemic (Hb < 110 g/l)	4264	62.3	58.2–65.5
Severely anemic (Hb < 70g/l)	283	4.1	3.4–5.9
Moderately anemic (HB 70–109 g/l)	3981	58.3	55.4–60.1
Normal (Hb > 110m/l)	2808	37.7	35.8–40.2
Ferritin Levels			
Ferritin deficient (<12 μg/l)	3361	47.1	30.7–35.4
Ferritin Normal (>12 μg/l)	3777	52.9	62.6–69.2
Vitamin A Levels			
Deficient (< = 0.70 μmol/l)	3694	52.6	49.6–55.1
Non deficient (>0.70 μmol/l)	3198	47.4	44.9–50.4
Zinc Levels			
Deficient (<60 μg/dl)	2625	38.8	36.2–41.1
Non-Deficient (60–150 μg/dl)	4166	61.2	58.9–63.7
Height for Age–Stunted			
Stunting (-6 to -2)	3061	44.5	43.7–48.1
Normal (-1.99 to +6)	3862	55.5	51.9–56.3
Weight for Height—Wasted			
Wasting (-5 to -2)	1050	15.5	14.1–16.8
Normal (-1.99 to +5)	5809	84.5	83.2–86.0
Weight for Age—Under weight			
Underweight (-6 to -2)	2315	33.6	32.6–36.5
Normal (-1.99 to +5)	4705	66.4	63.5–67.5
History of worm infestation			
Yes	597	8.3	7.5–9.9
No	6369	91.7	90.1–92.5

The univariate analysis (Table 3) showed that the IDA in children under five years of age in Pakistan was significantly associated with: age of less than 24 months (OR 1.45 1.29–1.62 p <0.05), stunting (OR 1.44 1.27–1.64 p<0.05), being underweight (OR 1.28 1.15–1.43 p<0.05), presence of clinical anemia (OR 5.03 4.41–5.74 p<0.05), history of worm infestation (OR 1.33 1.10–1.62 p<0.05), having a mother with IDA (OR 1.91 1.65–2.21 p<0.05) and household food insecurity (OR 1.23 1.08–1.41 p<0.05).

**Table 3:** Factors associated with Iron deficiency anemia among children 6–59 months of age in Pakistan, Univariate and Multivariate analysis (N = 2373).

Characteristics	IDA	(95% CI)	Un adjusted OR (95% CI)	P-value	Adjusted OR (95% CI)	P-value
**Household factors**						
Residence						
Rural	1423 (68.8)	65.2–72.3	0.89 (0.79–1.02)	0.088	0.77 (0.65–0.90)	0.001
Urban	950 (31.2)	27.7–34.8	Ref.			
Wealth quintiles						
Poorest	505 (22.3)	19.8–24.8	1.04 (0.86–1.26)	0.655	0.65 (0.51–0.85)	
2	448 (19.9)	17.9–21.9	1.06 (0.88–1.29)	0.545	0.83 (0.65–1.05)	
3	509 (20.9)	19.0–22.3	1.11 (0.91–1.35)	0.314	0.99 (0.79–1.26)	
4	510 (21.2)	19.2–23.3	1.26 (1.04–1.51)	0.017	1.19 (0.96–1.48)	
5 wealthiest	401 (15.6)	13.4–17.8	Ref.		Ref.	
Food security						
Food insecure	1460 (64.7)	62.2–67.1	1.23 (1.08–1.41)	0.003	1.20 (1.10–1.40)	0.002
Food secure	860 (35.3)	32.9–37.8	Ref.			
**Maternal factors**						
Maternal Education						
Illiterate	1293 (55.6)	52.7–58.4	1.11 (0.86–1.41)	0.439		
Primary or less (1–5)	378 (16.4)	14.6–18.3	1.41 (1.07–1.85)	0.016		
Middle(6–8)	218 (9.3)	7.8–10.7	1.40 (1.03–1.9)	0.032		
Matric(9–10)	288 (11.4)	9.8–12.9	1.28 (0.96–1.7)	0.09		
Intermediate & above (>10)	183 (7.4)	5.8–9.0	Ref.			
Maternal iron deficiency anema						
Anemic	558 (26.9)	24.6–29.3	1.91 (1.65–2.21)	0.000	1.72 (1.47–2.01)	0.001
Normal	1577 (73.1)	70.7–75.4	Ref.		Ref.	
BMI (kg/m2)						
<18.5	410 (17.5)	15.8–19.1	1.12 (0.97–1.29)	0.130		
18.5–24.9	1223 (52.5)	50.2–54.7	Ref.			
25–29.9	493 (20.1)	18.2–22.1	0.92 (0.79–1.04)	0.163		
> = 30	247 (9.9)	8.6–11.3	0.83 (0.69–0.99)	0.042		
**Child’s factors**						
Child’s sex						
Female	1067 (45.9)	43.5–48.4	0.86 (0.77–0.97)	0.012	0.87 (0.76–0.98)	0.028
Male	1306 (54.1)	51.6–56.5	Ref.		Ref.	
Child’s Age in months						
<24m	1252 (53.9)	51.5–56.2	1.45 (1.29–1.62)	0.000	1.35 (1.18–1.55)	0.0002
24-59m	1121 (46.1)	43.8–48.5	Ref.		Ref.	
Presence of clinical Anemia in children						
Yes	1229 (52.6)	49.7–55.6	5.03 (4.41–5.74)	0.000	5.69 (4.93–6.56)	0.0001
No	1131 (47.4)	44.4–50.3	Ref.		Ref.	
Vitamin A deficiency						
Deficient (< = 0.70 μmol/l)	1221 (51.5)	48.5–54.5	0.93 (0.83–1.05)	0.261		
Non deficient (>0.70 μmol/l)	1084 (48.5)	45.5–51.5	Ref.			
Zinc deficiency						
Deficient (<60 μg/dl)	852 (37.8)	34.9–40.6	1.07 (0.95–1.21)	0.240		
Non-Deficient (60–150 μg/dl)	1409 (62.2)	59.4–65.1	Ref.			
Height for Age–Stunted						
Stunting(-6 to -2)	1158 (50.4)	47.9–52.9	1.44 (1.27–1.64)	0.000	1.42 (1.23–1.63)	0.0001
Normal(-1.99 to +6)	1160 (49.6)	47.1–52.2	Ref.		Ref.	
Weight for Height–Wasted						
Wasting(-5 to -2)	351 (15.1)	13.5–16.7	0.95 (0.82–1.11)	0.532		
Normal(-1.99 to +5)	1963 (84.9)	83.3–86.5	Ref.			
Weight for Age—Under weight						
Underweight(-6 to -2)	868 (37.3)	35.0–39.6	1.28 (1.15–1.43)	0.000		
Normal(-1.99 to +5)	1481 (62.7)	60.4–65.0	Ref.			
History of worm infestation						
Yes	218 (9.8)	8.4–11.3	1.33 (1.1–1.62)	0.003		
No	2093 (90.2)	88.7–91.6	Ref.			

In the multivariate regression (Table 3) only being underweight was no longer a significantly increased risk with childhood IDA and of the variables that remained the adjusted odds ratios were slightly attenuated: age < 24 months (AOR 1.40, 95% CI 1.18–1.55 p <0.05), stunting (AOR 1.42 CI 1.23–1.63 p<0.05), having a mother with IDA (AOR 1.72 CI 1.47–2.01 p<0.05) and household food insecurity (AOR 1.20 CI 1.10–1.40 P<0.05), except for presence of clinical anemia for which the adjusted OR increased (AOR 5.69 CI 4.93–6.56 p<0.05). In contrast, living in a rural area (AOR 0.77 CI 0.65–0.90 p<0.05) and being a female child (AOR 0.87 CI 0.76–0.98 p<0.05) was associated with reduced odds of IDA (Table 3).

## Discussion

In our study, the prevalence of IDA in children aged 6–59 months was 33.2% which according to the WHO criteria represents a ‘moderate burden’ [2]. We also found a substantial prevalence of low hemoglobin levels, vitamin A deficiency, zinc deficiency, stunting, wasting, underweight and food insecurity amongst children aged 6–59 months living in Pakistan.

The prevalence of IDA in our study is lower than in previous studies in other low resource countries such as Palestine and Kenya [29–30] and substantially below the estimate from a previous study in Pakistan [14]. These differences could be attributed to variations in the study settings or factors such as the rate of parasitic infections and dietary habits. The previously published study from Pakistan, reported a prevalence of IDA of 63% from a smaller sample (n = 320) taken from a semi urban area, which is not comparable with this population based survey. Additionally it was conducted 18 years ago, before the introduction of the iron fortification in Pakistan [31]. Our findings are consistent with IDA prevalence reported in Kazakhstan (32.4%) [32], Yemen (34.2%) [33] and the Pakistan National Nutrition Survey (36%) undertaken in 2001 [13] but are higher than studies conducted in Morocco (20.4%) [34], India (23.1%) [35] and Iran (29.1%) [36].

Our study found that the food insecurity status of households is significantly associated with IDA in children, a finding that is consistent with the available literature examining this association [37, 38, and 39]. Food insecurity is characterized by either unavailability of food or inability to procure and access food and has consequences in both macro and micronutrient deficiencies. In Pakistan the widespread food insecurity situation reflects the economic instability of many areas of the country [40]. The study found a relationship between maternal iron deficiency anemia and IDA in children that confirms previous reports [18, 41–43] and highlights that IDA is common in pregnant and non-pregnant women of reproductive age in Pakistan [13]. In our study clinical examination of children detected clinical anemia in 53.6% of IDA cases. This sign can therefore assist in diagnosis where facilities for biochemical testing are not available.

We found the prevalence of IDA to be significantly associated with children’s age, with the youngest children having the highest odds of IDA. This finding is consistent with similar studies conducted in Iran, India and the Philippines [36, 39, 44]. The first two years of life is a period of rapid growth with an increased iron requirement therefore risk of IDA is increased in this age. Moreover factors such as limited access to iron rich food, inadequate infant and young child feeding practices including lack of exclusive breast feeding, prolonged breast feeding and inappropriate weaning food and recurrent illnesses increase the chance of young children developing IDA [17, 39, and 45]. We also found that the odds of IDA increased when the child was stunted and food insecure, suggesting that malnutrition is a contributing factor for IDA [9, 13, 46].

After adjusting for other factors, our study found that female children and those residing in rural areas were less likely to develop IDA. There is conflicting evidence with regard to the relationship between sex and IDA in children. Contrary to our study, data from Yemen and India found a higher prevalence of IDA in girls than boys [36, 47]; however studies from Western Kenya and Haiti found boys to be more at risk [48, 49]. In our setting children living in rural areas have more access to green leafy vegetables which may explain their reduced risk of IDA.

The major strength of this study is the nationally representative sample using reliable methods to detect iron and adjustment for confounding factors. However this study also has some limitations. The cross-sectional nature of the study means that the temporal relationship between IDA and the associated factors cannot be established. The information on various indicators was collected using a structured questionnaire that may have been prone to recall bias and presence of some missing data is also a potential limitation. Moreover we did not measure serum vitamin B12 and folate levels and so cannot measure other forms of anemia, suggesting some misclassification bias.

The data presented provides valuable insight into the prevalence of IDA in children which has remained fairly consistent in Pakistan over the last decade. Considering the burden of IDA in Pakistani children it is essential that interventions such as iron supplementation, food fortification and diet diversification should be used at scale [50], currently iron supplementation and wheat flour fortification are successful strategies being used worldwide. There is good evidence to suggest that iron supplementation improves hemoglobin level and reduces IDA prevalence in children [51–53]. Studies conducted in Pakistan also reveal iron supplementation to children and mothers results in improved iron stores [54–56]. Further a recent pooled analysis and studies in Central Asia, Venezuela and Iran suggest that wheat flour fortification can significantly improve iron status at the population level [54, 57–60] but there is a lack of such supplementation programs in Pakistan.

To reduce the burden of IDA, Pakistan needs a holistic approach of short term vertical programs such as iron supplementation and long term horizontal programs including wheat flour fortification. Specifically, further efforts should be made to restore the national wheat flour fortification program as wheat is the staple diet of Pakistan. Additional investments are also required to improve; education, especially for females, food insecurity through food supplements and agricultural support. These may require novel methods such as behavioral change communications to improve dietary patterns and infant and young child complementary feeding (IYCF) practices. Finally for this generation of Pakistani children to benefit it is important that Pakistan acts swiftly to alleviate the prevalence of IDA.

## References

[1] McLean E, Cogswell M, Egli I, Wojdyla D, De Benoist B. Worldwide prevalence of anaemia, WHO vitamin and mineral nutrition information system, 1993–2005. Public health nutrition. 2009 4 1;12(04):444–54. 18498676 10.1017/S1368980008002401

[2] WHO UNCF, UNU (2001) Iron deficiency anemia assessment, prevention, and control: A guide for program managers. Geneva.

[3] Badham J, Zimmermann MB, Kraemer K, editors. The guidebook nutritional anemia Basel, Switzerland: Sight and Life Press; 2007.

[4] WHO. Micronutrient Deficiencies- Iron Deficiency Anemia. WHO; Available: http://www.who.int/nutrition/topics/ida/en/. Accessed 28 September 2015.

[5] Grantham-McGregor S, Ani C. A review of studies on the effect of iron deficiency on cognitive development in children. The Journal of nutrition. 2001 2 1;131(2):649S–68S. 11160596 10.1093/jn/131.2.649S

[6] Lozoff B, Georgieff MK. Iron deficiency and brain development In Seminars in pediatric neurology 2006 9 30 (Vol. 13, No. 3, pp. 158–165). WB Saunders. 17101454 10.1016/j.spen.2006.08.004

[7] Black MM, Quigg AM, Hurley KM, Pepper MR. Iron deficiency and iron-deficiency anemia in the first two years of life: strategies to prevent loss of developmental potential. Nutrition reviews. 2011 11 1;69(suppl 1):S64–70. 10.1111/j.1753-4887.2011.00435.x 22043885

[8] Scott SP, Chen-Edinboro LP, Caulfield LE, Murray-Kolb LE. The impact of anemia on child mortality: an updated review. Nutrients. 2014 12 22;6(12):5915–32. 10.3390/nu6125915 25533005 PMC4277007

[9] Stoltzfus RJ. Defining iron-deficiency anemia in public health terms: a time for reflection. The Journal of nutrition. 2001 2 1;131(2):565S–7S. 11160589 10.1093/jn/131.2.565S

[10] Institute for Health Metrics and Evaluation. The Global Burden of Disease: Generating Evidence, Guiding Policy. Seattle, WA: IHME, 2013

[11] Balarajan Y, Ramakrishnan U, Özaltin E, Shankar AH, Subramanian SV. Anaemia in low-income and middle-income countries. The Lancet. 2012 1 6;378(9809):2123–35. 10.1016/S0140-6736(10)62304-5 21813172

[12] Hamedani P, Hashmi KZ, Manji M. Iron depletion and anaemia: prevalence, consequences, diagnostic and therapeutic implications in a developing Pakistani population. Current medical research and opinion. 1987 1 1;10(7):480–5. 3621993 10.1185/03007998709112407

[13] Pakistan Institute of Development Economics, Micro-nutrient Laboratories Aga Khan University and Medical Centre Karachi. National nutrition survey 2001–2002. Islamabad, Pakistan: Planning Commission, Government of Pakistan and UNICEF, 2004.

[14] Paracha PI, Hameed A, Simon J, Jamil A, Nawab G. Prevalence of Anaemia in Semi-Urban Areas of Peshawar, Pakistan-A Challenge for Health Professionals and Policy Makers. Journal-Pakistan medical association. 1997 2;47:49–53. 9071861

[15] Molla A, Khurshid M, Molla AM. Prevalence of iron deficiency anaemia in children of the urban slums of Karachi. Prevalence. 1992. 1507388

[16] Rahbar MH, Hozhabri S, Wang J. Prevalence of anaemia among children living in five communities in and near Karachi, Pakistan. Toxicological & Environmental Chemistry. 2007 4 1;89(2):337–46.

[17] Akhtar S, Ahmed A, Ahmad A, Ali Z, Riaz M, Ismail T. Iron status of the Pakistani population-current issues and strategies. Asia Pacific journal of clinical nutrition. 2013;22(3):340 10.6133/apjcn.2013.22.3.17 23945403

[18] Ahmed A, Ahmad A, Khalid N, David A, Sandhu MA, Randhawa MA, et al A question mark on iron deficiency in 185 million people of Pakistan: its outcomes and prevention. Critical reviews in food science and nutrition. 2014 12 2;54(12):1617–35. 10.1080/10408398.2011.645087 24580562

[19] National Institute of Population Studies (NIPS) [Pakistan] and ICF International. Pakistan demographic and health survey 2012–13 Demographic and Health Surveys. Islamabad, Pakistan, and Calverton, Maryland, USA: NIPS and ICF International, 2013

[20] World food program, A Manual: measuring and interpreting malnutrition and mortality. 2005. Available: http://www.unhcr.org/45f6abc92.pdf

[21] WHO (2009) Anthro Plus for personal computers manual: Software for assessing growth of the world’s children and adolescents. Available: http://www.who.int/growthref/tools/en. Accessed September 2015.

[22] Guyatt GH, Oxman AD, Ali M, Willan A, McIlroy W, Patterson C. Laboratory diagnosis of iron-deficiency anemia. Journal of general internal medicine. 1992 3 1;7(2):145–53. 1487761 10.1007/BF02598003

[23] Thurnham DI, McCabe LD, Haldar S, Wieringa FT, Northrop-Clewes CA, McCabe GP. Adjusting plasma ferritin concentrations to remove the effects of subclinical inflammation in the assessment of iron deficiency: a meta-analysis. The American journal of clinical nutrition. 2010 9 1:ajcn-29284. 10.3945/ajcn.2010.29284 20610634

[24] Grant FK, Suchdev PS, Flores-Ayala R, Cole CR, Ramakrishnan U, Ruth LJ, et al Correcting for inflammation changes estimates of iron deficiency among rural Kenyan preschool children. The Journal of nutrition. 2012 1 1;142(1):105–11. 10.3945/jn.111.146316 22157541 PMC4697949

[25] WHO. Haemoglobin concentrations for the diagnosis of anaemia and assessment of severity Vitamin and Mineral Nutrition Information System. Geneva, World Health Organization, 2011 (WHO/NMH/NHD/MNM/11.1). Available: http://www.who.int/vmnis/indicators/haemoglobin.pdf

[26] Pasricha SR, Flecknoe-Brown SC, Allen KJ, Gibson PR, McMahon LP, Olynyk JK, et al Diagnosis and management of iron deficiency anaemia: a clinical update. Med J Aust. 2010 11 1;193(9):525–32. 21034387 10.5694/j.1326-5377.2010.tb04038.x

[27] Coates J, Swindale A, Bilinsky P. Household Food Insecurity Access Scale (HFIAS) for measurement of food access: indicator guide Washington, DC: Food and Nutrition Technical Assistance Project, Academy for Educational Development 2007 8.

[28] IBM Corp. Released 2010. IBM SPSS Statistics for Windows, Version 19.0. Armonk, NY: IBM Corp.

[29] Sirdah MM, Yaghi A, Yaghi AR. Iron deficiency anemia among kindergarten children living in the marginalized areas of Gaza Strip, Palestine. Revista brasileira de hematologia e hemoterapia. 2014 4;36(2):132–8. 10.5581/1516-8484.20140030 24790539 PMC4005512

[30] Onimawo IA, Ukegbu PO, Asumugha VU, Anyika JU, Okudu H, Echendu CA, et al Assessment of anaemia and iron status of school age children (aged 7–12 years) in rural communities of Abia state, Nigeria. African Journal of Food, Agriculture, Nutrition and Development. 2010;10(5).

[31] Govt of Pakistan, a report on Nutritional interventions in Pakistan. Available: http://pc.gov.pk/mtdf/8-Nutrition/8-Nutrition.pdf

[32] Hashizume M, Kunii O, Sasaki S, Shimoda T, Wakai S, Mazhitova Z, et al Anemia and iron deficiency among schoolchildren in the Aral Sea region, Kazakhstan. Journal of tropical pediatrics. 2003 6 1;49(3):172–7. 12848209 10.1093/tropej/49.3.172

[33] Al-Zabedi EM, Kaid FA, Sady H, Al-Adhroey AH, Amran AA, Al-Maktari MT. Prevalence and risk factors of iron deficiency anemia among children in Yemen. American Journal of Health Research. 2014;2(5):319–26.

[34] El Hioui M13, Ahami AO, Aboussaleh Y. Iron deficiency and anaemia in rural school children in a coastal area of Morocco. Pakistan Journal of Nutrition. 2008;7(3):400–3.

[35] Jyothsna K, Madhavi S, Nagaveni D, Narayan D, Meka R. Anemia, iron deficiency, meat consumption, and hookworm infection in women of reproductive age in rural area in Andhra Pradesh. Ann Biol Res. 2011;2(3):209–16.

[36] Keikhaei B, Zandian K, Ghasemi A, Tabibi R. Iron-deficiency anemia among children in southwest Iran. Food and nutrition bulletin. 2007 12 15;28(4):406–11. 18274167 10.1177/156482650702800405

[37] Skalicky A, Meyers AF, Adams WG, Yang Z, Cook JT, Frank DA. Child food insecurity and iron deficiency anemia in low-income infants and toddlers in the United States. Maternal and child health journal. 2006 3 1;10(2):177–85. 16328705 10.1007/s10995-005-0036-0

[38] Park K, Kersey M, Geppert J, Story M, Cutts D, Himes JH. Household food insecurity is a risk factor for iron-deficiency anaemia in a multi-ethnic, low-income sample of infants and toddlers. Public health nutrition. 2009 11 1;12(11):2120–8. 10.1017/S1368980009005540 19405987

[39] Pasricha SR, Black J, Muthayya S, Shet A, Bhat V, Nagaraj S, et al Determinants of anemia among young children in rural India. Pediatrics. 2010 7 1;126(1):e140–9. 10.1542/peds.2009-3108 20547647

[40] Kugelman M, editor. Hunger Pains: Pakistan's Food Insecurity. Woodrow Wilson International Center for Scholars; 2010.

[41] Kumar A, Rai AK, Basu S, Dash D, Singh JS. Cord blood and breast milk iron status in maternal anemia. Pediatrics. 2008 3 1;121(3):e673–7. 10.1542/peds.2007-1986 18310187

[42] Singla PN, Tyagi M, Shankar R, Dash D, Kumar A. Fetal iron status in maternal anemia. Acta Paediatrica. 1996 11 1;85(11):1327–30. 8955460 10.1111/j.1651-2227.1996.tb13919.x

[43] Sweet DG, Savage G, Tubman TR, Lappin TR, Halliday HL. Study of maternal influences on fetal iron status at term using cord blood transferrin receptors. Archives of Disease in Childhood-Fetal and Neonatal Edition. 2001 1 1;84(1):F40–3. 11124923 10.1136/fn.84.1.F40 PMC1721190

[44] Tengco LW, Rayco-Solon P, Solon JA, Sarol JN Jr, Solon FS. Determinants of anemia among preschool children in the Philippines. Journal of the American College of Nutrition. 2008 4 1;27(2):229–43. 18689554 10.1080/07315724.2008.10719695

[45] Charles CV. Iron deficiency anemia: a public health problem of global proportions. Public health–methodology, environmental and systems issues. 2012:109.

[46] Desalegn A, Mossie A, Gedefaw L. Nutritional iron deficiency anemia: magnitude and its predictors among school age children, southwest ethiopia: a community based cross-sectional study. PloS one. 2014 12 1;9(12):e114059 10.1371/journal.pone.0114059 25438147 PMC4250059

[47] Kaur IP, Kaur S. A comparison of nutritional profile and prevalence of anemia among rural girls and boys. Journal of Exercise Science and Physiotherapy. 2011 6;7(1):11.

[48] Foote EM, Sullivan KM, Ruth LJ, Oremo J, Sadumah I, Williams TN, et al Determinants of anemia among preschool children in rural, western Kenya. The American journal of tropical medicine and hygiene. 2013 4 3;88(4):757–64. 10.4269/ajtmh.12-0560 23382166 PMC3617865

[49] Bernard S. Prevalence and risk factors of anemia among children 6–59 Months Old in Haiti. Anemia. 2013 Mar 10;2013. 10.1155/2013/502968 PMC3608103 23555053

[50] Pasricha SR, Drakesmith H, Black J, Hipgrave D, Biggs BA. Control of iron deficiency anemia in low-and middle-income countries. Blood. 2013 4 4;121(14):2607–17. 10.1182/blood-2012-09-453522 23355536

[51] Gera T, Sachdev HP, Nestel P, Sachdev SS. Effect of iron supplementation on haemoglobin response in children: systematic review of randomised controlled trials. Journal of pediatric gastroenterology and nutrition. 2007 4 1;44(4):468–86. 17414146 10.1097/01.mpg.0000243440.85452.38

[52] Sylvetsky AC, Jefferds ME, De‐Regil LM, Dowswell T. Intermittent iron supplementation for improving nutrition and developmental outcomes in children. The Cochrane Library. 2011. 10.1002/14651858.CD009085.pub2 PMC4547491 22161444

[53] Pasricha SR, Hayes E, Kalumba K, Biggs BA. Effect of daily iron supplementation on health in children aged 4–23 months: a systematic review and meta-analysis of randomised controlled trials. The Lancet Global Health. 2013 8 31;1(2):e77–86. 10.1016/S2214-109X(13)70046-9 25104162

[54] Soofi S, Cousens S, Iqbal SP, Akhund T, Khan J, Ahmed I, et al Effect of provision of daily zinc and iron with several micronutrients on growth and morbidity among young children in Pakistan: a cluster-randomised trial. The Lancet. 2013 7 12;382(9886):29–40. 10.1016/S0140-6736(13)60437-7 23602230

[55] Nisar YB, Dibley MJ. Antenatal iron–folic acid supplementation reduces risk of low birthweight in Pakistan: secondary analysis of Demographic and Health Survey 2006–2007. Maternal & child nutrition. 2016 1 1;12(1):85–98. 25422133 10.1111/mcn.12156 PMC6860143

[56] Bhutta ZA, Rizvi A, Raza F, Hotwani S, Zaidi S, Hossain SM, et al A comparative evaluation of multiple micronutrient and iron–folic acid supplementation during pregnancy in Pakistan: impact on pregnancy outcomes. Food and nutrition bulletin. 2009 12 25;30(4 suppl4):S496–505. 20120791 10.1177/15648265090304S404

[57] Das JK, Salam RA, Kumar R, Bhutta ZA. Micronutrient fortification of food and its impact on woman and child health: a systematic review. Systematic reviews. 2013 8 23;2(1):1. 23971426 10.1186/2046-4053-2-67 PMC3765883

[58] Tazhibayev S, Dolmatova O, Ganiyeva G, Khairov K, Ospanova F, Oyunchimeg D, et al Evaluation of the potential effectiveness of wheat flour and salt fortification programs in five Central Asian countries and Mongolia, 2002–2007. Food and nutrition bulletin. 2008 12 15;29(4):255–65. 19227050 10.1177/156482650802900402

[59] Layrisse M, García-Casal MN, Méndez-Castellano H, Jiménez M, Olavarría HC, Chávez JF, et al Impact of fortification of flours with iron to reduce the prevalence of anemia and iron deficiency among schoolchildren in Caracas, Venezuela: a follow-up. Food and nutrition bulletin. 2002 12 1;23(4):384–9. 16619746 10.1177/156482650202300412

[60] Sadighi J, Mohammad K, Sheikholeslam R, Amirkhani MA, Torabi P, Salehi F, et al Anaemia control: lessons from the flour fortification programme. Public health. 2009 12 31;123(12):794–9. 10.1016/j.puhe.2009.09.024 19914671

